# Learning about microbial language: possible interactions mediated by microbial volatile organic compounds (VOCs) and relevance to understanding *Malassezia* spp. metabolism

**DOI:** 10.1007/s11306-021-01786-3

**Published:** 2021-04-07

**Authors:** Andrea Rios-Navarro, Mabel Gonzalez, Chiara Carazzone, Adriana Marcela Celis Ramírez

**Affiliations:** 1 Cellular and Molecular of Pathogenic Microorganisms Research Group (CeMoP), Biological Sciences Department, Universidad de Los Andes, Cra 1 No. 18A-12, Bogotá, 111711 Cundinamarca Colombia; 2 Laboratory of Advanced Analytical Techniques in Natural Products (LATNAP), Chemistry Department, Universidad de Los Andes, Cra 1 No. 18A-12, Bogotá, 111711 Cundinamarca Colombia

**Keywords:** Volatile organic compounds (VOCs), *Malassezia*, Lipid metabolism, Fungal diseases, Interaction processes, Microbial volatiles

## Abstract

**Background:**

Microorganisms synthesize and release a large diversity of small molecules like volatile compounds, which allow them to relate and interact with their environment. Volatile organic compounds (VOCs) are carbon-based compounds with low molecular weight and generally, high vapor pressure; because of their nature, they spread easily in the environment. Little is known about the role of VOCs in the interaction processes, and less is known about VOCs produced by *Malassezia*, a genus of yeasts that belongs to the human skin mycobiota. These yeasts have been associated with several dermatological diseases and currently, they are considered as emerging opportunistic yeasts. Research about secondary metabolites of these yeasts is limited. The pathogenic role and the molecular mechanisms involved in the infection processes of this genus are yet to be clarified. VOCs produced by *Malassezia* yeasts could play an important function in their metabolism; in addition, they might be involved in either beneficial or pathogenic host-interaction processes. Since these yeasts present differences in their nutritional requirements, like lipids to grow, it is possible that these variations of growth requirements also define differences in the volatile organic compounds produced in *Malassezia* species.

**Aim of review:**

We present a mini review about VOCs produced by microorganisms and *Malassezia* species, and hypothesize about their role in its metabolism, which would reveal clues about host-pathogen interaction.

**Key scientific concepts of review:**

Since living organisms inhabit a similar environment, the interaction processes occur naturally; as a result, a signal and a response from participants of these processes become important in understanding several biological behaviors. The efforts to elucidate how living organisms interact has been studied from several perspectives. An important issue is that VOCs released by the microbiota plays a key role in the setup of relationships between living micro and macro organisms. The challenge is to determine what is the role of these VOCs produced by human microbiota in commensal/pathogenic scenarios, and how these allow understanding the species metabolism. *Malassezia* is part of the human mycobiota, and it is implicated in commensal and pathogenic processes. It is possible that their VOCs are involved in these behavioral changes, but the knowledge about this remains overlocked. For this reason, VOCs produced by microorganisms and *Malassezia* spp. and their role in several biological processes are the main topic in this review.

## What are volatile organic compounds and where do they come from?

It is important to know what VOCs are and why they are indispensable in understanding metabolic processes in *Malassezia* and other microorganisms. VOCs are carbon-based compounds with low molecular weight and generally, high vapor pressure, and for these reasons, they spread easily in the environment (Bennet & Inamdar, 2015; Schmidt et al. 2015). The aroma of several familiar smells like cheese, wine, foodstuffs, yogurt, even human odor as well as repugnant smells are from microbial volatiles (Kai et al. 2009; Veselova et al. 2019), but knowing its biogenic source is harder. For this reason, it is important to unravel the original source of VOCs to understand their role in metabolic and interactional processes.

**Fig. 1:**
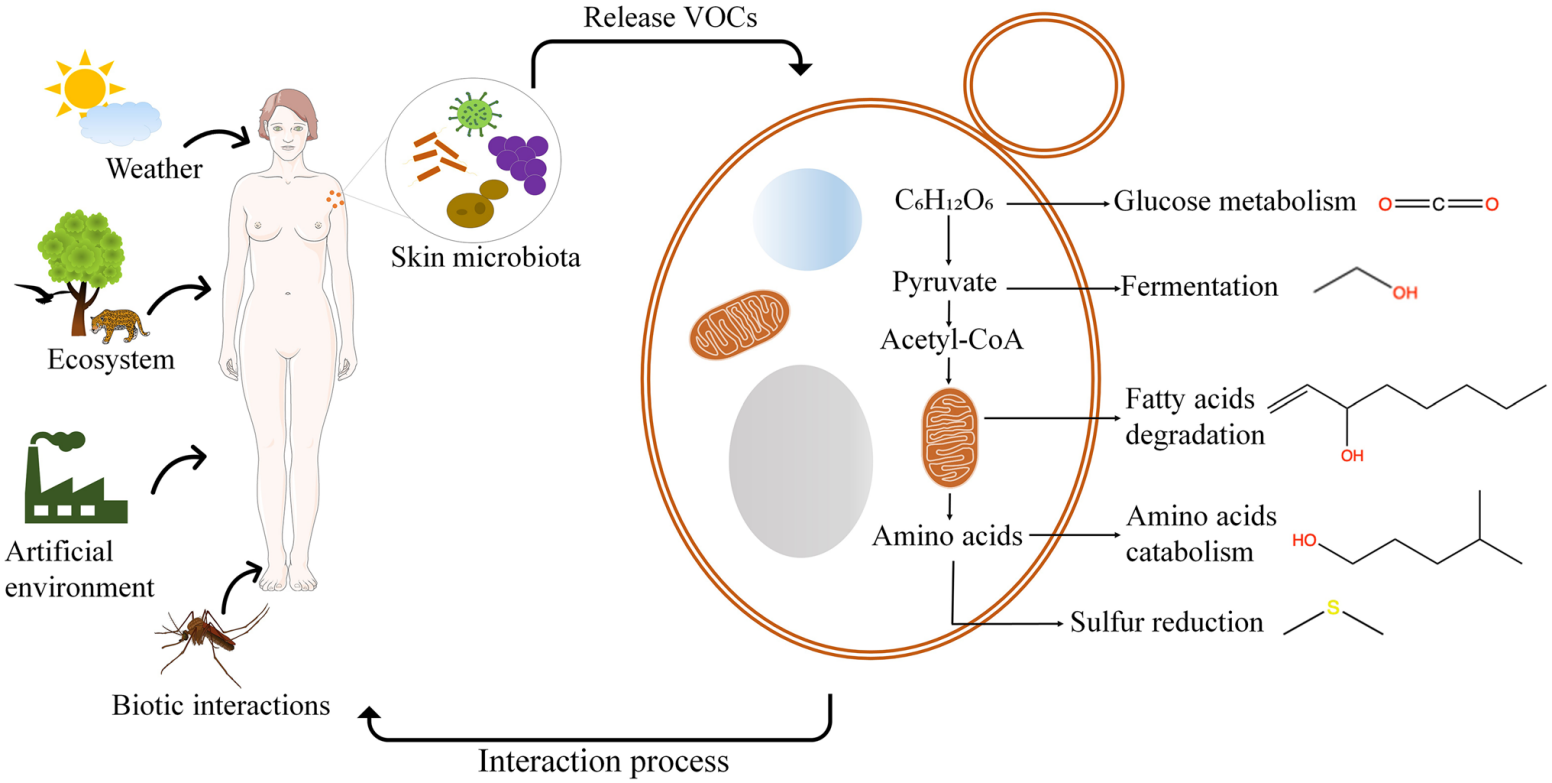
VOCs biogenesis. Several environmental factors influence skin microbiota. As a result, microorganisms release volatile that are synthesized by different metabolism pathways. Thus, the main compound produced in the glucose metabolism is carbon dioxide. During the fermentation processes release ethanol, and fatty acid degradation produces 1-octen-3-ol. Finally, amino acids catabolism and sulfur reduction, release 3- methylbutane-1-ol and dimethyl sulfide respectively. These VOCs are involved in interaction processes between skin microbiota and the environment. This figure was created using some image from Servier Medical Art Commons Attribution 3.0 Unported License. (http://smart.servier.com). Servier Medical Art by Servier is licensed under a Creative Commons Attribution 3.0 Unported License

Microorganisms from diverse environments release a wide range of VOCs due to their metabolism, but the exact biosynthesis pathways remain unclear. This suggests that VOCs originate from both central and secondary metabolism (Schmidt et al. 2015). Some metabolic processes by which VOCs are produced involve glucose metabolism, amino acids catabolism, fermentation, fatty acids degradation and sulfur reduction (Peñuelas et al. 2014). Figure 1 shows the possible pathways that could produce VOCs.

Among the VOCs released by microorganisms, it is common to find alkenes, alcohols, ketones and other hydrocarbon compounds with C6‒C16 chains, which derive from the intermediates of fatty acids metabolism, including β-oxidation (Peñuelas et al. 2014). Decarboxylation reactions result in the formation of alkanes, 1-alkenes, and methyl ketones, while the reduction of the carboxyl group produces aldehydes and aliphatic alcohols (Peñuelas et al. 2014; Schulz & Dickschat, 2007). Sulfur-based organic compounds are released by a wide range of microorganisms, a common compound found in this group is dimethyl sulfide. Sulfur derivates like dimethyl disulfide, dimethyl trisulfide and methanethiol are produced because of methionine cleavage (Veselova et al. 2019). Glucose is the main energy source of many microorganisms, and volatiles, such as carbon dioxide, is released by its degradation (Peñuelas et al. 2014). Pyruvate, lactate and acetate are intermediates of the glycolysis process and act as precursors to VOCs, such as ethanol and other alcohols, as well as acidic compounds (Audrain et al. 2015). As a result of amino acids metabolism, keto acids, such as 2-oxoglutarate, oxaloacetate, pyruvate, 2-oxoisovalerate, 2-oxoisocaproate and 2-oxo-2- methylvalerate, are produced. These keto acids are synthesized by deamination of the amino acids glutamate, aspartate, alanine, valine, leucine and isoleucine. The subsequent decarboxylation and reduction reactions convert these acids into the corresponding aldehydes, ketones and alcohols (Audrain et al. 2015; Peñuelas et al. 2014; Veselova et al. 2019). Almost every metabolic pathway result in the production of VOCs, which are important in the interaction processes between microorganisms, organisms and the environment (Effmert et al. 2012; Wenke et al. 2010; Wielkopolan & Obrępalska-Stęplowska, 2016).

In addition, there are several factors involved in the production and release of VOCs, such as the species that the VOCs originate from, the substrates, the amount of radiation, associations with other microorganisms, or the type of ecosystem and climate (Bennett et al. 2012). Moreover, the composition of VOCs produced by the microbial population depends on the diversity of species and environmental conditions, like the availability of carbon energy sources, pH and temperature (Duffy & Morrin, 2019). According to the ecological niches and metabolic differences between microbes, it is important to consider these factors in determining microbial volatiles.

Many applications of VOCs are found in industrial products such as paint thinners, air fresheners, dry-cleaning fluids, and as well in other industrial activities in modern society (Bennett & Inamdar, 2015). Despite this, volatiles from plants and bacteria have received greater attention due to their applications in biotechnological and industrial processes, rather than fungi VOCs, which is lagging scientific knowledge (Piechulla & Degenhardt, 2014; Schulz & Dickschat, 2007). Since microbial volatiles have become of greater scientific interest due to their biological/ecological potential, researchers have consolidated information concerning microbial volatiles, and the mVOCs database was created. There are 1860 volatile compounds registered to both bacteria and fungi; however, VOCs belonging to *Malassezia* species have not been registered in this database (Lemfack et al. 2014, 2018).

## VOCs produced by bacteria/fungi and their role in interactional processes

Fungi are organisms with a dynamic metabolism and, just like bacteria, they produce many volatile compounds with a lot of possible applications which have a biological/ecological impact, as shown by previous studies. For example, some fungal species can produce a unique profile of VOCs, which differ depending on growth conditions (Bennett & Inamdar, 2015; Bennett et al. 2012). Presently, research is far from understanding the profile of VOCs in *Malassezia*; as a result, little is known about its secondary metabolism. In this study, we present a compilation of the information about VOCs from *Malassezia* spp. and other related microorganisms to understand the possible role of these compounds in the conversion from commensal to pathogenic behavior.

Although VOCs in *Malassezia* are not well known, other microorganisms are better understood. Volatiles from bacteria and fungi have different fields of application like bioprospection, industrial and clinical applications (Juarez et al. 2020; Kanchiswamy et al. 2015; Morath et al. 2012; Sethi et al. 2013). There are many types of microbial interactions like bacteria-bacteria, bacteria-fungi, fungi-fungi, fungi-bacteria, bacteria-plant, fungi-plant, and all of them determine behavioral responses which facilitate the communication and development of the microorganisms and organisms involved (Schulz-Bohm et al. 2017). Most of the interactions studied are those that involved bacteria. To survive, bacteria can produce an antagonistic response that helps it to defend against others; it has been shown that the release of dimethyl disulphide from *Serratia plymuthica* inhibits the growth of *Agrobacterium tumefaciens* and *Agrobacterium vitis.* These findings postulate antagonistic bacteria as a biocontrol alternative to plant pathogens (Dandurishvili et al. 2011). Another bacteria used as biocontrol is *Bacillus amyloliquefaciens,* as its volatile compounds suppress the biofilm formation and production of antioxidants of the tomato pathogen *Ralstonia solanacearum* (Raza et al. 2016). Moreover, a study showed the antiproliferative activity of 52 VOCs produced by bacteria, standing out γ-Lactones, which interfere with the quorum-sensing-systems of bacteria (Schulz et al. 2010). An additional example of the effect of VOCs on bacterial growth is the study that demonstrated that compound dimethylhexadecylamine produced by bacteria with a plant growth–promoting activity affected the viability and motility of *Arthrobacter agilis*, *Bacillus* sp. and *Pseudomonas* sp. (Martínez-Cámara et al. 2019).

There are studies that show the type of interactions between bacteria-fungi since VOCs produced by bacteria also affect the growth and survival of fungi. For instance, VOCs from *Bacillus subtilis* can cause alterations in pathogenic fungi such as *Fusarium oxysporum, Paecilomyces lilacinus, and Alternaria alternata,* by the induction of structural malformations such as mycelial and conidial deformation (Chaurasia et al. 2005). Moreover, it is known that VOCs emitted from antagonistic bacteria like *Bacillus*, *Burkholderia*, *Pseudomonas*, *Serratia*, *Staphylococcus* and *Stenotrophomonas* species negatively influence the mycelial growth of the soil-borne phytopathogenic fungus *Rhizoctonia solani* (Kai et al. 2007). On the other hand, the synergic activity of VOCs from *Pseudomonas aeruginosa* over *Aspergillus fumigatus* has been demonstrated. This study suggests that dimethyl sulfide stimulates the growth of the fungus, which has implications in the progress of coinfection by these human pathogens (Briard et al. 2016). A further consequence of interaction is the negative effects between microorganisms. VOCs from *Enterobacter aerogenes* inhibit the growth of *Saccharomyces cerevisiae* increasing intracellular reactive oxygen species (ROS) (Wu et al. 2019). Another bacteria whose VOCs exhibit adverse effects on fungi is *Rahnella aquatilis;* its volatiles inhibit the mycelial formation of *Colletotrichum gloeosporioides*, also bacterial volatiles increase the permeability of the cell membrane of this fungus and downregulate the expression of pathogenicity-related genes during mycelial infection (Kong et al. 2020).

Some VOCs produced by fungi also have antibacterial activity. A type of interaction between fungi-bacteria shows the mechanisms of how the VOCs of *Muscodor albus* inhibit bacteria *Escherichia coli*. Its VOCs produce DNA damage, disruption of DNA repair and alteration of the response to stress. For this organism, it has been suggested that VOCs induce alkylation (Alpha et al. 2015). Another fungus which has antagonistic effects on bacteria and fungi is *Ganoderma pfeifferi*; its volatile compounds produce antimicrobial activity against *Staphylococcus aureus*, *Bacillus subtilis*, *Escherichia coli*, and antifungal effect against *Candida albicans* (Al-Fatimi et al. 2016). In addition, fungal VOCs can change the expression profile of bacteria. This is the case of *Fusarium culmorum.* Its volatiles produce changes in gene and protein expression related to motility, signal transduction, energy metabolism and secondary metabolite production of *Serratia plymuthica* (Schmidt et al. 2017).

VOCs are also important in fungi-fungi interaction. A previous study showed that the profile of VOCs changed during the interaction between four wood rooting fungi (*Bjerkandera adusta*, *Hypholoma fasciculare*, *Stereum gausapatum* and *Trametes versicolor*). In this study, the volatile profile was different in the combinations of growth tested. Moreover, all the species, except *T. versicolor*, produced at least five specific VOCs (Evans et al. 2008). A different case of this type of interaction is the antagonistic relationship between a yeast and a fungus*.* VOCs, specifically benzyl alcohol produced by *Starmerella bacillaris*, reduce mycelial growth of *Botrytis cinerea*. This finding is relevant in the biocontrol of plant pathogens (Lemos et al. 2020). Similar findings demonstrated that volatiles produced by *Trichoderma longibrachiatum* inhibit the growth of plant-pathogens fungi *Sclerotium rolfsii* and *Macrophomina phaseolina* through the alteration of mycelial structure (Sridharan et al. 2020). Since there is little information about fungal volatiles, further research is necessary to elucidate the role of fungal VOCs in biological/ecological processes.

Volatile organic compounds produced by microorganisms are not only important due to interactional processes carried out between them but also because VOCs are mediating signals between other organisms related to the microbial host. Studies about the interaction type microbe-host-insect have been conducted. It has been reported that VOCs from bacteria belonging to human microbiota like *Staphylococcus* sp., *Corynebacterium* sp., *Bacillus* sp., produce certain volatiles that attract malaria mosquitoes (Verhulst et al. 2009, 2010), and they have been reported also in the yeast *Malassezia furfur* (Gonzalez et al. 2019). Further analyzes are necessary to elucidate the role of these VOCs for *Malassezia* in the interaction between fungi-host-insect. Similar findings were reached by Tabares et al. in which the behavioral response of *Rhodnius prolixus* to VOCs produced by bacteria from human facial skin was evaluated. The authors demonstrated that VOCs produced by bacteria such as *Staphylococcus capitis*, *Staphylococcus warneri* and *Staphylococcus epidermidis* were attractive to *R. prolixus*, while the VOCs released by *Citrobacter koseri*, *Brevibacterium epidermidis* and *Micrococcus luteus* were non-attractive (Tabares et al. 2018). Since insects interact with odorants emitted by plants, fungi, and other volatile sources, and these olfactory signals are involved in aggregation behavior or serve in oviposition stimulants. Volatiles also are important in host location and attraction to food sources (Davis et al. 2013; Hung et al. 2015). It is necessary to conduct studies to unravel the importance of VOCs released by human microbiota in biotic interactions. To sum up, these approaches might provide clues for elucidating the biological and ecological significance of microbial VOCs emissions and will help to unravel the importance of volatiles in biological interactions between microbes and their hosts. Table 1 shows microbial volatiles and their possible associated roles.

**Table 1:** Some representative VOCs originated from microorganisms and their putative role in interaction processes

VOCs	Microorganism	Possible biological function	Reference
Alcohols (ethanol and larger alkanols)	*Muscodor albus*	Membrane disruption/dissipation of the proton gradient	(Alpha et al., 2015)
3-Methylbutanal	Methicillin-susceptible *Staphylococcus aureus* (MSSA)	Not reported	(Ashrafi et al., 2018)
Pentanal			
2-Methylpropan-1-ol	MSSA, *Pseudomonas aeruginosa*	Biofilms	
3-Methylbutan-1-ol			
Nonan-2-one	*Pseudomonas aeruginosa*	Biofilms	
5-Methylhexan-2-amine			
5-Methylheptan-2-amine			
Undecan-2-one			
Ethanol			
Butan-2-ol	*Streptococcus pyogenes*	Not reported	
Undec-1-ene		Biofilms	
Dimethyl disulfide	*Pseudomonas aeruginosa, Streptococcus pyogenes*	Biofilms, microbial interaction	
2,5-Dimethylpyrazine	*Pseudomonas aeruginosa*	Not reported	(Briard et al., 2016)
Octan-3-one			
(3E)-Penta-1,3-diene	Mushroom	Fruit odor	(Combet et al., 2006)
2-Methylpropan-2-ol	*Aspergillus sp.*	No reported	(Gerritsen et al., 2018)
2-Methylfuran			
2-Methylpropan-1-ol			
2-Methylbutan-2-ol			
2-Ethylhexan-1-ol			
1,2,7,7-Tetramethylbicyclo [2.2.1] heptan-2-ol			
(3E)-Penta-1,3-diene			
1-Methyl-4-propan-2-ylcyclohexa-1,4-diene	*Fusarium culmorum*	Bacterial motility	(Schmidt et al., 2016)
3-Methylidene-6-propan-2-ylcyclohexene			
3,7,7-Trimethylbicyclo [4.1.0] hept-3-ene			
Sesquiterpenes	*Trichoderma* spp.	Antagonist to *Laccaria bicolor*	(Guo et al., 2019)
Monoterpenes/sesquiterpenes	*Flammulina velutipes*	Atractive to mites	(Li et al., 2018)
Oct-1-en-3-ol	*Trametes gibbosa*	Atractive for fungus-eating beetles	(Drilling & Dettner, 2009)
2-Mmethylbutan-1-ol	*Trichoderma longibrachiatum*	Signaling molecules in microbe-microbe interactions and antimicrobial activity	(Sridharan et al., 2020)
Terphenyl-2-ol			
1-Acetylazepan-2-one			
Isobutyl acetate farnesene			
Trimethyl pentanol			

## VOCs and human applications

Microbial volatiles are used by humans to improve their daily activities in several fields like diagnosis of diseases, agriculture and/or bioprospecting. Volatiles produced by microorganisms can be used as a biomarker of diseases. A recent review summarized the evidence demonstrating that exhaled breath is a non-invasive way to diagnose microbial infection, as VOCs produced by microorganisms can represent a fingerprint to disease; for example, exhaled nitric oxide is related to asthmatic airway inflammation, and this compound has been used in clinical diagnosis; other infections of the respiratory tract where the VOCs produced by pathogens useful for diagnosis are: pneumonia, pulmonary aspergillosis, pulmonary tuberculosis and influenza (Ahmed et al. 2017). Moreover, it has been reported that different microorganisms release volatiles specifically associated with a type of infection (Elmassry & Piechulla, 2020). In addition, there is evidence to postulate VOCs from feces as biomarkers of *Clostridium difficile* (formerly *Clostridiodes difficile*) infection (Patel et al. 2019). A study of volatile signatures that could be used in the identification of *Pseudomonas aeruginosa* was performed with 24 *P. aeruginosa* clinical isolates, where they identified 391 non-redundant compounds. Of these, only 70 were common in all isolates (Bean et al. 2017). Furthermore, *Aspergillus*, in isolates from fluid of patients with invasive pulmonary aspergillosis (IPA), produced a distinct volatile profile, offering possible IPA markers through these VOCs (Gerritsen et al. 2018). An additional study about *A. fumigatus*, the causal agent of invasive aspergillosis (IA), reported its volatile profile in vitro, which showed that the monoterpenes camphene, α-pinene, β-pinene, and limonene, and the sesquiterpenes α-trans-bergamotene and β-transbergamotene were representative compounds. Also, they studied the breath of patients with and without IA, and they found that the terpenoid ketone trans-geranylacetone and a β-vatirenene–like sesquiterpene distinguished patients with IA from patients without IA (Koo et al. 2014). Thus, this evidence has been useful to expand diagnostic tools using VOCs as biomarkers (Bean et al. 2017; Broza et al. 2014). One more case of microbial volatiles used as a diagnostic tool is the non-invasive diagnosis of infected cutaneous wounds through VOCs sampling from biofilms produced by bacteria as an in vitro model (Ashrafi et al. 2018). Surprisingly, a novel study showed that volatiles from sebum acts as biomarkers in Parkinson’s disease. Compounds such as hippuric acid, eicosane and octadecanal were found to be differential in patients in relation to disorder mentioned. This outcome suggests a possible role of species belonging to microbiota, like *Malassezia* spp., whose lipid-dependent metabolism could trigger the overproduction of volatiles from fatty acids pathway and producing effects in this disease (Trivedi et al. 2019).

In the agriculture industry, VOCs produced by harmless microorganisms have been used to control plant pathogens (Alpha et al. 2015). For example, *Trichoderma* spp. releases volatile compounds which can inhibit the growth of plant-pathogens during the interaction with *Laccaria bicolor* (Guo et al. 2019). VOCs are used also in this field, where volatiles from bacteria and fungi could be used to protect plants from pathogens. 1-octen-3-ol produced by many fungi enhances plant resistance to *Botrytis cinerea* (Kanchiswamy et al. 2015). In addition, *Starmerella bacillaris* is a yeast that acts as a potential biocontrol for gray mold on apples, since VOCs from *S. bacillaris* inhibit 90% of mycelial growth (Lemos et al. 2020). Moreover, volatiles released by rhizobacterium *Proteus vulgaris* increased the fresh weight of *Arabidopsis thaliana* through an interplay between the auxin and cytokinin pathways (Bhattacharyya et al. 2015). In addition, it has been shown that volatiles from beneficial plant fungi such as *Ampelomyces* sp. and *Cladosporium* sp. reduce diseases in *Arabidopsis* plant by *Pseudomonas syringae.* Moreover, VOCs promote growth in plants (Naznin et al. 2014). It would be worthwhile to elucidate the bioactivity of these compounds in *Malassezia* and postulate them as bioprospecting compounds.

## Why is it important to study VOCs produced by *Malassezia* spp.?

*Malassezia* yeasts are lipophilic and lipid dependent. They are commensal microorganisms found in human and animal skin (Grice & Dawson, 2017). However, *Malassezia* becomes a health concern when it acts opportunistically, suggesting that under certain conditions, the establishment of dermatologic and systemic diseases in the host could be triggered (Pedrosa et al. 2014). Recently, *Malassezia* has been associated with Crohn’s disease (Limon et al. 2019) and Parkinson’s disease (Laurence et al. 2019). Currently, there are 18 species of this genus in the class Malasseziomycetes, phylum *Basidiomycota* (Lorch et al. 2018). Some of them have been involved in several dermatological pathologies, such as pityriasis versicolor, seborrheic dermatitis/dandruff, and atopic dermatitis (Nowicka & Nawrot, 2019). Some species of *Malassezia* have been connected to fungemia and opportunist infections (Wu et al. 2015). Although the mechanisms involved in human diseases are not clear, it has been suggested that certain conditions are required in the host for the pathogenic processes to occur. For instance, changes in temperature, humidity, fat content in sebaceous glands, sweat, immune response, and even the presence of other microorganisms can be considered triggering factors (Theelen et al. 2018).

This peculiar yeast behavior may be related to its metabolic incapacity to synthesize de novo C12 or C16 fatty acid due to the absence of genes encoding fatty acid synthase (Celis et al. 2017). For this reason, all *Malassezia* species require an exogenous source of lipids to fulfill their growth requirements. Thus, this yeast is forced to live in the sebaceous areas of its host, such as the scalp, back, face and chest (Grice & Dawson, 2017). To do this, *Malassezia* intakes lipids from its host through the secretion of lipases, phospholipases, and sphingomyelinases (Wu et al. 2015); these enzymes are involved in the hydrolysis of triglycerides, releasing fatty acids indispensable to the yeast. A slight imbalance in this process could result in skin disorders which are mediated by immune response (Gordon et al. 2013).

Despite the efforts to understand all metabolic bases concerning *Malassezia* genus, many things are still unknown. For example, which factors influence the lipids’ metabolism? What VOCs are produced because of metabolism? How are VOCs involved in the host–pathogen relationship? Thus, there are many questions to answer concerning *Malassezia* behavior. Since the understanding of the metabolism of lipids in these yeasts is relevant to clarify their commensal/pathogenic behavior, two different studies have been performed to demonstrate their dynamic metabolism. First, through in silico metabolic network reconstruction of the lipid-synthesis pathways in *Malassezia* species, it was possible to prove that there are differences in the production of steroids in *M. furfur* and in the metabolism of butanoate in *M. pachydermatis*; moreover, the predictions obtained by these metabolic reconstructions suggested defects in the assimilation of palmitic acid in *M. globosa*, *M. sympodialis*, *M. pachydermatis*, and the atypical variant of *M. furfur*. Also, this study allowed for the detection of differences in the lipid-assimilation between *Malassezia* species (Triana et al. 2017). For example, *M. furfur* usually assimilates different kinds of tween including tween 20, 40, 60, and 80 while *M. globosa* and *M. sympodialis* do not assimilate any tween, and atypical *M. furfur* only assimilate tween 80. Additionally, it has demonstrated that oleic acid is fungistatic for *M. furfur*, *M. sympodialis* and atypical *M. furfur*, but when these strains are cultivated on a mixture of oleic acid and palmitic acid, the growth is increased (Table 2). It is not clear which mechanisms underlie these fungistatic effects. It has suggested that individual fatty acids create an imbalance in the lipidic composition comprising the cell membrane, and therefore, their survival (Celis, 2017; Triana et al. 2017). Secondly, a study about differences in the lipidome of *Malassezia* spp. was conducted, demonstrating the metabolic versatility in the species tested. The authors found differences in the proportion of cholesteryl ester and other lipids in *M. furfur*, atypical *M. furfur*, and *M. pachydermatis*. Also, uncommon lipids in yeast, like diacylglyceryltrimethylhomoserine and fatty acid esters of hydroxyl fatty acids (FAHFA), were found in a variable concentration in these *Malassezia* species (Celis et al. 2020). However, information about volatiloma of *Malassezia* species remains to be clarified.

**Table 2:** Lipid assimilation for some *Malassezia* species cultivated in minimal medium (MM)

Strain	T20	T40	T60	T80	PA	OA	PA + OA
*M. furfur*	+ +	+ +	+ +	+ +	±	-	+ +
Atypical *M. furfur*	-	-	-	+ +	-	±	+ +
*M. pachydermatis*	±	±	±	±	-	-	-
*M. sympodialis*	-	-	-	-	-	-	±
*M. globosa*	-	-	-	-	-	-	nd

T20: Tween 20; T40: Tween 40; T60: Tween 60; T80: Tween 80; PA: Palmitic acid; OA: Oleic acid; nd: not discernible. – no growth; ± weak growth; +  + good growth (Triana et al., 2017; Celis, 2017)

Currently, the study of VOCs produced by *Malassezia* has become a matter of interest because they could be involved, or most likely participate in biotic interactions like *Malassezia*-bacteria, *Malassezia*-fungi, *Malassezia*-host-vectors, and pathogenic processes as described for other microorganisms (Briard et al. 2016; Martínez-Cámara et al. 2019; Tyagi et al. 2020). Also, the VOCs could give important insights into the intriguing metabolic aspect of this yeast (Scotter et al. 2005).

## VOCs produced by *Malassezia* spp. How much do we know?

Lipid-dependence from *Malassezia* represents a major part of its metabolism involving the use of the fatty acid from the host, and many sub-products are released, like volatile compounds. Two studies were developed which tried to clarify the composition of VOCs produced by *Malassezia.* The first study was conducted in 1979 when *Malassezia* still belonged to *Pityrosporum* genus (Labows et al. 1979). In this study, the authors discovered for the first time that this yeast can produce VOCs on lipid supplemented media. Moreover, lipids such as oleic acid, triolein or human sebum, stimulate VOCs production in *Malassezia* species. Using gas chromatography-mass spectrometry (GC–MS), Labows et al. identified VOCs from four *Pityrosporum* species: *P. ovale, P. canis, P. pachydermatis and P. orbiculare*-currently, *M. furfur*, *M. pachydermatis* and *M. globosa* respectively (Gaitanis et al. 2012)- in solid media. Among the 11 compounds detected in all species, γ-lactones were the most representative. Six γ-lactones appeared in different proportions depending on the growth medium used; for example, in *P. ovale* (*M. furfur*) grown in Sabouraud-dextrose agar (SDA) with human sebum (1%) was characterized by the production of γ -lactones. Moreover, the volatile profile obtained from the growth in yeast nitrogen base agar (YNBA) supplemented with lecithin resulted in lactones such as γ-hexalactone and γ-heptalactone. However, as these experiments changed species and the type of medium simultaneously, it is difficult to determine the significance of separating each variable which determines the VOCs profile. However, lactones appear as a particular group of *Malassezia.* In contrast in comparison with other species like *Candida albicans* or *Saccharomyces cerevisiae*, lactones were not detected using the same media, but isopentanol and 2-phenylethanol were identified (Labows et al. 1979). These same authors mention that the highest recovery of volatiles occur when solid rather than liquid growth media are used; this could be explained because these microorganisms prefer a specific place in the host that provides them with the necessary sources to grow and produce VOCs. Lactones may play an important role in scalp odors and could be involved in characteristic human odors (Labows et al. 1979). The study above mentioned was the first to identify VOCs in these yeasts. However, there was little information about them, and the topic was overlooked.

The metabolic pathway to produce lactones in yeast and fungi are well described. In brief, there are four main metabolic routes to produce lactones: hydroxylation, β-oxidation and lactonisation of fatty acids; reduction of unsaturated lactones; Baeyer—Villiger oxidation of cyclic ketone and α, ω-oxidation of alkanes or fatty acids. Due to the importance of lipids in *Malassezia* metabolism, hydroxylation, β-oxidation and lactonisation of fatty acids could be involve in volatile production in this genus. The first step in this pathway is the hydrolysis of fatty acids given by enzymatic processes, after having a hydrolyzed fatty acid, β-oxidation occurs, which takes place in the peroxisome, presenting four reactions that oxidize the substrate until it is in shorter chains (C12 to C8), finally, isomerization and lactonization happens, where there are several reactions that mainly depend on the microorganism and the environmental conditions; in this step, the lactone structure is generated according to the position in which the hydroxyl group remains forming γ-, δ- or ε-lactone. Additionally, it is important to mention that the intake of fatty acids by fungi is determined by the interactions of the fungal cell wall and the substrate, having the ability to modify their surface properties in the presence of hydrophobic substrates to facilitate their intake (Romero-Guido et al. 2011).

Recently, a study in *Malassezia* genus revealed the production of 61 VOCs in different growth media: mDixon, and minimal medium supplemented with oleic acid, oleic acid + palmitic acid, and palmitic acid for *M. furfur*; the analyses were conducted in exponential and stationary phases of the yeast (Gonzalez et al. 2019). The compounds were characterized by headspace solid-phase microextraction (HS-SPME) and gas chromatography-mass spectrometry (GC–MS). Alkanes, alcohols, ketones, furanic compounds and some previously reported γ-lactones for *Malassezia*, were identified. In this study, a chemical differentiation of the compounds in all treatments were gathered. For example, the γ-dodecalactone was detected in the mDixon and the oleic acid medium in both growth phases, while the hexan-1-ol was found in all treatments, except for the palmitic acid medium. This suggests that *Malassezia* VOCs production is stimulated by the compounds in the growth media, demonstrating once again the dynamic metabolism in this yeast. Also, the results showed the decrease in volatile production in a medium supplemented with palmitic acid in which the alkanes were predominant (Gonzalez et al. 2019).

The fact that in the first study was possible to detect diversity of lactones, and in the recent research only found γ-dodecalactone may be due to differences between the experimental conditions in each study. For instance, there are genetic differences among the tested strains, because in 1979, they used the strains ATCC 24047, 12078, and 14521 of *M. furfur*, in contrast, in 2019 they used *M. furfur* CBS 1878. Other reasons are the differences in the growth media, and mainly the techniques employed for VOCs sampling. However, the different growing requirements of species, combined with the importance of media determining VOCs suggest secondary metabolites also differ between species (Gonzalez et al. 2019).

Additionally, a common compound characterized between fungi and bacteria is dimethyl sulfide, present in *M. fufur* too. This has been related to the stimulation of *A. fumigatus* growth in pathogenic conditions (Briard et al. 2016). These findings are confirmed in a study that showed sulfur compounds produced by *P. aeruginosa* when infecting *Galleria mellonella* promoting the growth of *A. fumigatus* in a coinfection condition, so a synergistic increase in mortality and of fungal and bacterial burdens are evident in this animal model. This interaction between pathogen microorganisms could be explicated by the metabolism of *A. fumigatus*. It has been suggested that this fungus assimilates sulfur compounds released by *Pseudomonas* via cysteine or homocysteine synthase pathways (Scott et al. 2019). It could be useful to conduct research to see if this compound has the same role in *Malassezia* and can be postulated as a marker of microbial interactions. Other compounds identified were 3 methylbutan-1-ol and methylpropan-1-ol, which came from amino acid metabolism (Bjurman, 1999). The participation of fatty acid in central metabolism of *Malassezia* spp. is known. Compounds such as alkanes and ketones identified in *M. furfur* like pentan-2-one, undecan-2-one arise from fatty acid metabolism (Bjurman, 1999). Further analysis could explain the role or possible applications of these VOCs in *Malassezia* species.

Due to the lipid-dependence of *Malassezia* species (Wu et al. 2015), their metabolisms through enzyme-type lipases could be an essential pathway to produce VOCs. Although VOCs from *Malassezia* are not fully understood, previous studies suggest these yeasts exhibit metabolic differences according to growth conditions; for instance, it has demonstrated that there are differences in the lipidome species which include fatty acids (Celis et al. 2020). Furthermore, metabolic modeling demonstrated controversy in the synthesis and the assimilation of fatty acids by *Malassezia* (Triana et al. 2017). We hypothesize that the composition in lipids of *Malassezia* may produce different VOCs because the use of lipids results in the release of specific compounds. Further investigations are needed to prove this hypothesis.

## Perspectives

Since *Malassezia* could act as a pathogen for humans and animals, it is important to evaluate the mechanisms by the yeast triggered diseases. We need to combine lipidomic analysis with the volatilome analysis to comprehend the metabolic pathways involved in VOCs synthesis and its importance in determining the conversion between commensal and pathogenic stages of *Malassezia* species. VOCs have become interesting to elucidate host-interaction processes, and even they could have biotechnological applications. Moreover, it is indispensable to research the relationship between fatty acids and VOCs since fatty acids are precursors in the reaction of eight-carbon volatile synthesis and are key components in a variety of lipids (Combet et al. 2006).

## Conclusion

Microorganisms including *Malassezia* spp. produce volatile organic compounds in their secondary metabolism, and these VOCs are important in interactional/biological processes. Further investigation should be conducted to fully characterize volatile compounds found in *Malassezia* species, and then evaluate their role in beneficial/pathogenic and interaction processes. With of more complete picture of VOCs, it will be possible to postulate alternatives to control diseases and set up potential bioprospecting of these yeasts.
